# Erratum: Han, D.-H.; Kang, L.-H. Terahertz Displacement and Thickness Sensor with Micrometer Resolution and Centimeter Dynamic Range

**DOI:** 10.3390/s20061605

**Published:** 2020-03-13

**Authors:** Dae-Hyun Han, Lae-Hyong Kang

**Affiliations:** 1 Department of Mechatronics Engineering, and LANL-JBNU Engineering Institute-Korea, Jeonbuk National University, 567 Baekje-daero, Duckjin-gu, Jeonju-si, Jeonbuk 54896, Korea; dh.han@jbnu.ac.kr; 2 Department of Mechatronics Engineering, Department of Flexible and Printable Electronics, and LANL-JBNU Engineering Institute-Korea, Jeonbuk National University, 567 Baekje-daero, Duckjin-gu, Jeonju-si, Jeonbuk 54896, Korea

The authors wish to make the following erratum to this paper [1].

Funding is incorrect and must be replaced by the following Funding:

**Funding:** This material is based upon work supported by the Ministry of Trade, Industry & Energy (MOTIE, Korea) under the Sensor Industry Enhancement Program (20003125, Development of an ultraprecision measurement sensor based on the fiber composite for flaw detection of the composite structure), and the Korea Institute for Advancement of Technology (P0007067).

The authors would like to apologize for any inconvenience caused to the readers by these changes.
